# Non- pancreatic neuroendocrine tumour presenting with hypoglycemia in an elderly patient

**DOI:** 10.4314/ahs.v20i4.44

**Published:** 2020-12

**Authors:** Merve Eren, Feyzi Bostan

**Affiliations:** Department of Internal Medicine, University of Health Sciences Antalya Training and Research Hospital, Antalya

**Keywords:** neuroendocrine tumour, hypoglycaemia, prednisone, non-islet cell tumour

## Abstract

**Background:**

Hypoglycemia is a common, symptom seen in individuals. Hypoglycemia in the elderly is both under-recognized and misdiagnosed due to nonspesific hypoglycemic symptoms and accompanying comorbidities in this population. In diabetic individuals, hypoglycemia is most commonly caused by administering insulin or sulphonylureas and insulin secretagogues. Other drugs, such as antibiotics or beta-blockers, have been reported to reduce blood glucose to abnormally low levels. Hypoglycemia in non-diabetic patients is considered a rare event, and the possible reasons may be reactive hypoglycemia, insulin-secreting tumours and other malignancies, hypopituitarism, hypocortisolism, alcohol abuse, inappropriate insulin self-administration, malnutrition, renal failure and sepsis.

**Case:**

An 86- year- old male was admitted to the emergency department with hypoglycemia diagnosed with non-pancreatic neuroendocrine tumour (NET) on lung secreting insulin. No surgical intervention or chemotherapy was planned due to patients age and comorbidities so best supportive care was planned. We used prednisone for symptomatic treatment of hypoglycemia and the patient has been followed up periodically. In this period he had no hypoglycemic attack.

**Conclusion:**

For patients with hypoglycemia who are unable/decline to receive any further treatment, low dose glucocorticoid is a good choice to achieve normoglycemia. It seems to be more cost effective compared to other treatment options. Furthermore hospitalisation rates may decrease due to decreased hypogylcemic attacks.

## Introduction

Hypoglycemia is a common, under-recognized symptom seen in individuals. Elderly individuals are more vulnerable to complications of hypoglycemia than others because of co-morbidities and senility. Furthermore, autoregulatory responses are impaired through aging, which means the symptoms of hypoglycemia are often less specific and are therefore either missed or incorrectly diagnosed as transient ischemic attacks or other cerebrovascular events. The elderly are also more prone to the effects of hypoglycemia such as the increased risk of accidents, falls, fractures, hospitalizations, in-hospital mortality and long-term impairment ocognition. Thus it is very important to evaluate the patient for hypoglycemia when there is a clinical suspicion.

The most common endogenous cause of hypoglycemia is hyperinsulinemia secondary to islet cell tumours of the pancreas. 1 Hypoglycemia due to non-pancreatic tumours is infrequently reported. 2 Ectopic insulin secretion has been reported in few cases but not convincingly proved. Treatment of hypoglycemia due to insulin secreting tumours has two parts. One is symptomatic treatment of hypoglycemia till curative treatment of insulin secreting tumour by surgery or medical agents and second is curative treatment of the tumour. There are some medications for symptomatic treatment of hypoglycemia. One involves glucocorticoids which are more feasible and cost effective than other medications such as somatostatin analogues. Here we report a case o af non-pancreatic neuroendocrine tumour (NET) on lung secreting insulin for whom we used prednisone for symptomatic treatment of hypoglycemia.

## Case presentation

**Figure 1:**
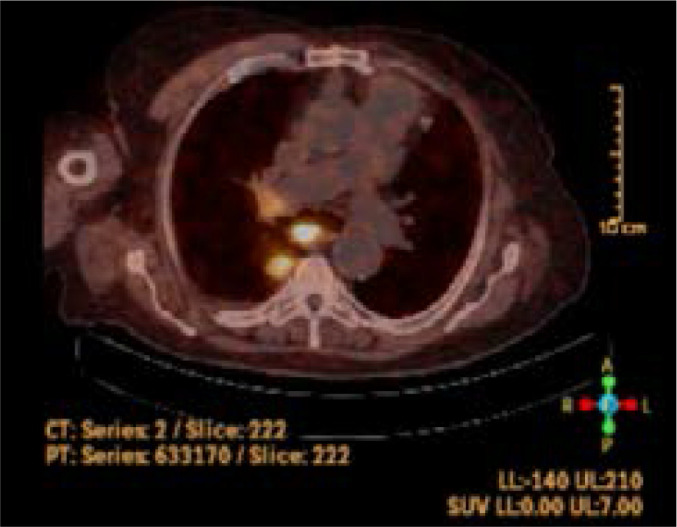
Ga-68 DOTATATE PET/CT scan image of the lesion, axial view

**Figure 2:**
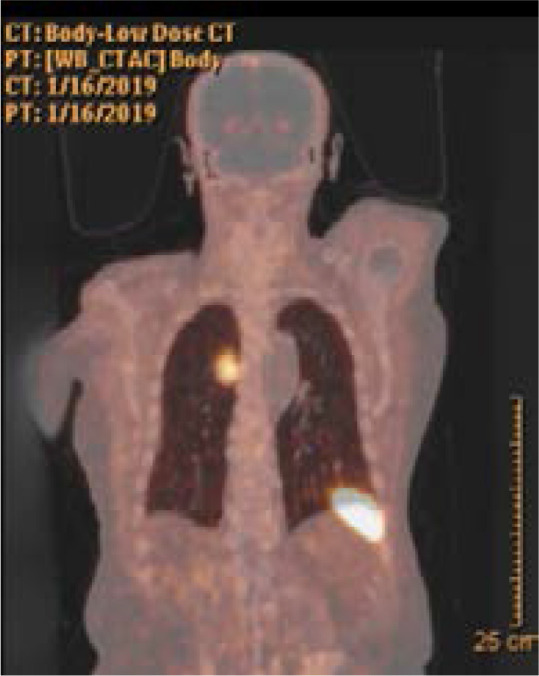
Ga-68 DOTA TATE PET/CT scan image of the lesion, coronal view

An 86- year- old male was admitted to the emergency department with altered mental status. He had a similar episode nine months ago and was told that this situation was due to hypoglycemia but no further tests were done. He had a history of coronary bypass surgery, myocardial infarction, heart failure, systemic hypertension, chronic obstructive lung disease but no diabetes. He had not been prescribed insulin or oral hypoglycemic agents before. No one in the household was diabetic or using any medication for diabetes. He was immobile for two years due to vertebral fracture. His vital signs were stable (pulse rate 70/min, blood pressure 110/70 mmHg, respiratory rate 14/min, oxygen saturation: 96% on room air and afebrile). He was agitated, disoriented and uncooperative on physical examination. His neurological examination was normal. His venous glucose level was 34 mg/dl (N: 74 – 106 mg/dl), his laboratory values were otherwise unremarkable on admission. He was admitted to the internal medicine department for further evaluation of hypoglycemia. His capillary glucose level was monitored and intravenous dextrose infusion planned when hypoglycemia was detected. His plasma glucose level was 53 mg/dl (N: 74 – 106 mg/dl), insulin was 5.21 uIU/ml (N: 1.9 – 23 uIU/mL), c- peptide was 3.5 ng/ml (N: 0.8 – 4.2 ng/ml). Even though these values were in normal ranges for our hospital laboratory, they were consistent with endogenous hyperinsulinemia according to Endocrine Society Guideline. 2 Abdominal ultrasound and CT scan were insignificant for any pathology. His pituitary, adrenal and thyroid hormone levels were in normal ranges. During further evaluation, low dose predinose (5 mg, twice a day) was started for symptom control and withdrawal of intravenous dextrose infusion. The patient did not have any hypogylcemic event since then. Ga-68 DOTATATE PET/CT scan was performed to look for any focus secreting endogenous insulin. This imaging revealed multiple mediastinal lymph nodes and a lesion on the superior segment of the right lower lobe, close to the pleura with a size of 20x27mm, surrounded with milimetric nodular densities associated with groundglass nodules at subpleural space. Figures 1, 2

A CT – guided biopsy from this lesion was performed by invasive radiologists and histopathology revealed high grade neuroendocrine carcinoma. The patient was referred to the medical oncology department; they did not suggest any surgical intervention or chemotherapy due to his advanced age and comorbidities and recommended supportive care. The patient has been followed up by our internal medicine outpatient clinic periodically. He died of decompensated heart failure after 3 months. He had used prednisone 10 mg/day for one month and 5mg/day thereafter. In this period he had no hypoglycemic attack.

## Discussion

In diabetic individuals, hypoglycemia is most commonly caused by administering insulin or sulphonylureas and insulin secretagogues. Other drugs, such as antibiotics or beta-blockers, have occasionally been reported to reduce blood glucose to abnormally low levels. 3–6 Hypoglycemia in non-diabetic patients is considered a rare event, occurring in less than 1% of all hospitalizations.^7^ Reported causes of hypoglycemia in non-diabetic patients include reactive hypoglycemia, insulin-secreting tumours and other malignancies, hypopituitarism, hypocortisolism, alcohol abuse, inappropriate insulin self-administration, malnutrition, renal failure and sepsis.^8^ – ^11^

Initial evaluation of hypoglycemia should proceed according to usual practice. The Endocrine Society Guidelines recommend investigation in patients in whom Whipple's triad is fulfilled.^2^ When performing laboratory investigation of hypoglycemia, it is imperative to draw a plasma glucose level not just a finger-stick capillary blood glucose level to confirm hypoglycemia with simltaneous measurement of levels of insulin, proinsulin, C-peptide, β-hydroxybutyrate and an oral hypoglycemic agent screening. 2 Such an approach evaluates and differentiates endogenous hyperinsulinism insulinoma, post-gastric bypass hypoglycemia, insulin autoimmune hypoglycemia, and accidental or surreptitious insulin secretagogue ingestion.

Malignancies causing hypogylcemia can be divided into several categories according to underlying mechanisms: tumour insulin secretion pancreatic islet b-cell tumours, insulinomas or non-islet-cell tumours such as bronchial carcinoid or gastrointestinal stromal tumours; tumour IGF2 precursors secretion (big IGF2) ‘IGF2-oma’ (leiomyosarcoma, fibrosarcoma); tumour somatostatin secretion ‘Somatostatinoma’ (pancreatic neuroendocrine tumour); tumour IGF1 secretion ‘IGF1-oma’ (large-cell carcinoma of the lung); tumour glucagon-like peptide 1 (GLP1) secretion ‘GLP1-oma’ (ovarian neuroendocrine tumour); autoantibodies to insulin or its receptor ‘Tumour autoimmune hypoglycemia’; or other tumour-related factors including massive tumour burden, massive liver tumour infiltration or pituitary and/or adrenal glands tumour destruction.^12^ Hypoglycemia caused by tumours other than insulinomas are usually referred to as ‘non-islet cell tumour hypoglycemia (NICTH). 13 NICTH should be suspected in any patient with hypoglycemia without clear etiology.

If there are clues to NICTH (eg, known malignancy, identification of large new mass), this can be pursued early. Otherwise, we would consider this rare diagnosis if the workup of the preceding causes was unrevealing.

Initial treatment of hypoglycemia is accomplished by oral glucose and/or iv glucose or dextrose-containing fluids as necessary. In many cases, this suffices to avoid further hypoglycemia. Once NICTH is identified and a primary tumour is found, the mainstay of treatment is surgical resection, which is curative for hypoglycemia if resection is complete. 14 However, in rare cases, total resection is either delayed or not feasible. Reasons for nonresectability include: large tumour burden, widely metastatic disease, compromised local structures necessitating subtotal resection, physical characteristics of the tumour and/or its relationship to surrounding structures necessitating abortion of resection and patient preference. In patients with NICTH who are not candidate for curative treatment, glucocorticoids are choices of therapeutic agents for symptomatic treatment.^15^ In this setting, there is no clear “standard of care.” Nutritional approaches may provide treatment from hypoglycemia but often do not suffice. Local or systemic targeted antitumour therapy may be an option for some patients and may be successful. In cases where this is not available or does not free the patient from hypoglycemia, the literature suggests the careful use of glucocorticoids. 16 Glucocorticoids have been successfully used as “bridge” therapy to resection. 15,17, 18 Complete freedom from iv dextrose using glucocorticoid monotherapy in nonresectable cases was also reported.^15^,^19^ – ^23^

In our case an elderly patient experiencing altered consciousnes due to hypoglycemia was diagnosed with high grade neuroendocrine carcinoma. He was suitable neither for any chemotherapy nor surgical intervention because of his accompanying comorbidities and patients' preference. For such patients with hypoglycemia who are unable/decline to receive any further treatment, low dose glucocorticoid is a good choice to achieve normoglycemia. It seems to be more cost effective method compared to other treatment options. Furthermore hospitalisation rates may decrease due to decreased hypogylcemic attacks.
